# Policy and Science for Global Health Security: Shaping the Course of International Health

**DOI:** 10.3390/tropicalmed4020060

**Published:** 2019-04-10

**Authors:** Kavita M. Berger, James L. N. Wood, Bonnie Jenkins, Jennifer Olsen, Stephen S. Morse, Louise Gresham, J. Jeffrey Root, Margaret Rush, David Pigott, Taylor Winkleman, Melinda Moore, Thomas R. Gillespie, Jennifer B. Nuzzo, Barbara A. Han, Patricia Olinger, William B. Karesh, James N. Mills, Joseph F. Annelli, Jamie Barnabei, Daniel Lucey, David T. S. Hayman

**Affiliations:** 1 Gryphon Scientific, LLC, 6930 Carroll Avenue, Suite 810, Takoma Park, MD 20912, USA; margaret@gryphonscientific.com; 2 Disease Dynamics Unit, Department of Veterinary Medicine, University of Cambridge, Madingley Road, Cambridge CB3 0ES, UK; jlnw2@cam.ac.uk; 3 Brookings Institution, 1775 Massachusetts Avenue NW, Washington, DC 20036, USA; bonniedjenkins@gmail.com; 4 Women of Color Advancing Peace, Security and Conflict Transformation, 3695 Ketchum Court, Woodbridge, VA 22193, USA; 5 Rosalynn Carter Institute for Caregiving, Georgia Southwestern State University, 800 GSW State University Drive, Americus, GA 31709, USA; jenolsen.drph@gmail.com; 6 Department of Epidemiology, Mailman School of Public Health, Columbia University, 722 West 168th St., New York, NY 10032, USA; ssm20@cumc.columbia.edu; 7 Ending Pandemics and San Diego State University, San Diego, CA 92182, USA; lgresham@sdsu.edu; 8 U.S. Department of Agriculture, National Wildlife Research Center, Fort Collins, CO 80521, USA; Jeff.Root@aphis.usda.gov; 9 Institute for Health Metrics and Evaluation, Department of Health Metrics Sciences, University of Washington, 2301 Fifth Avenue, Suite 600, Seattle, WA 98121, USA; pigottdm@uw.edu; 10 Wellcome Centre for Human Genetics, Nuffield Department of Medicine, University of Oxford, Roosevelt Drive, Oxford OX3 7BN, UK; 11 Next Generation Global Health Security Network, Washington, DC 20001, USA; t.winkleman.dvm@gmail.com; 12 RAND Corporation, 1200 South Hayes St., Arlington, VA 22202, USA; 13 Population Biology, Ecology, and Evolution Program, Emory University, Atlanta, GA 30322, USA; thomas.gillespie@emory.edu (T.R.G.); wildlifedisease@gmail.com (J.N.M.); 14 Department of Environmental Health, Rollins School of Public Health, 1518 Clifton Road, Atlanta, GA 30322, USA; 15 Center for Health Security, Johns Hopkins University School of Public Health, Pratt Street, Baltimore, MD 21202, USA; jnuzzo1@jhu.edu; 16 Cary Institute of Ecosystem Studies, Box AB Millbrook, NY 12545, USA; hanb@caryinstitute.org; 17 Environmental, Health and Safety Office (EHSO), Emory University, 1762 Clifton Rd., Suite 1200, Atlanta, GA 30322, USA; patty.olinger@emory.edu; 18 EcoHealth Alliance, 460 West 34th Street, New York, NY 10001, USA; karesh@ecohealthalliance.org; 19 Practical One Health Solutions, LLC, New Market, MD 21774, USA; pohsolutions@gmail.com; 20 Plum Island Animal Disease Center, Department of Homeland Security, Greenport, NY 11944, USA; jbarnabei87@gmail.com; 21 Department of Medicine Infectious Disease, Georgetown University, 600 New Jersey Avenue, NW Washington, DC 20001, USA; daniel.lucey8@gmail.com; 22 EpiLab, Infectious Disease Research Centre, School of Veterinary Science, Massey University, Private Bag, 11 222, Palmerston North 4442, New Zealand

**Keywords:** One Health, zoonoses, Ebola virus, emerging infectious diseases

## Abstract

The global burden of infectious diseases and the increased attention to natural, accidental, and deliberate biological threats has resulted in significant investment in infectious disease research. Translating the results of these studies to inform prevention, detection, and response efforts often can be challenging, especially if prior relationships and communications have not been established with decision-makers. Whatever scientific information is shared with decision-makers before, during, and after public health emergencies is highly dependent on the individuals or organizations who are communicating with policy-makers. This article briefly describes the landscape of stakeholders involved in information-sharing before and during emergencies. We identify critical gaps in translation of scientific expertise and results, and biosafety and biosecurity measures to public health policy and practice with a focus on One Health and zoonotic diseases. Finally, we conclude by exploring ways of improving communication and funding, both of which help to address the identified gaps. By leveraging existing scientific information (from both the natural and social sciences) in the public health decision-making process, large-scale outbreaks may be averted even in low-income countries.

## 1. Introduction

For decades, researchers have been studying infectious diseases affecting people, domestic and wild animals, and plants. Researchers have characterized emerging infectious diseases from viruses such as Human Immunodeficiency Virus (HIV) [1] and Severe Acute Respiratory Syndrome (SARS) coronavirus (CoV) [2,3], and bacteria such as *Escherichia coli* O104:H4 in Germany and France [4,5]. Approximately 75% of emerging pathogens have their origins in non-human reservoir hosts and are classic examples of zoonoses [6]. Furthermore, antimicrobial resistance among zoonotic diseases has become a significant health security challenge [7,8,9]. Combined with vaccine research and development (R&D) and immunization campaigns, scientific studies have contributed to the prevention or reduction of disease transmission globally [10,11,12]. Existing scientific knowledge and experience could be built upon to prevent or mitigate future outbreaks. However, under pressure to respond quickly to emerging outbreaks, decision-makers struggle to identify effective and relevant medical and non-medical public health response measures because they may not have available information about the causative agents, assessments of potential health and/or economic effects, effective biosafety and infection control measures, information about societally appropriate control measures, and ready risk communication measures for their constituents. Three primary types of gaps (data and models, safety and security, and cultural awareness) limit the translation of research findings in the decision-making process before, during, and after emergencies.

The 2014–2016 West-African Ebola virus disease (EVD) outbreak reinforced the concept that a major pathogen outbreak in one country can affect other countries throughout the region and world, and highlighted the aforementioned gaps in leveraging existing knowledge and practices to facilitate outbreak response [13,14]. This outbreak demonstrated that urban settings, socio-cultural traditions, and local migration affect outbreak dynamics. These lessons, along with the development and use of an experimental Ebola virus vaccine, contributed to very different responses in the 2018 outbreaks in the Democratic Republic of Congo (DRC) [15]. However, conflict and an unsafe public health response environment in the DRC towards the end of 2018 and into 2019 have led to a significant increase of known cases to over 1000 [16]. As long as the security situation ensues, the number of cases will continue to increase and the ability of researchers to collect information about circulating strains will be hampered.

In addition, advancing genomic sequencing capabilities are used to generate increasing amounts of data about bacteria, viruses, and other microorganisms in different locations. For example, the U.S. government has supported sequencing and modelling studies to identify different strains of pathogens in nature and evaluate their potential to initiate or drive outbreaks of local and international concern. The Canadian government, World Health Organization, U.S. government, non-governmental organizations (e.g., ProMED-mail), private companies, and research groups have leveraged data analytics platforms to analyze these and other available data and attempt to identify potential outbreaks before they become significant public health problems [17,18,19,20]. These platforms integrate epidemiological or syndromic data from a variety of sources, both official (e.g., Ministry of Health reports) and unofficial (e.g., media reports) sources, to help identify potential outbreaks as early as possible. The utility of these and related efforts relies on access to data, the sharing of which is governed by different international and national-level policies, and on awareness among policy-makers that scientific information, however uncertain, can inform initial and ongoing assessments of infectious disease risk and response [21,22]. These platforms do not appear to incorporate systematically the results from environmental scanning, modeling, and other related research fields. These platforms vary by the purpose, their intended stakeholders, the data they integrate, their analytic capabilities and methodologies, their accuracy, and other factors, all of which have different utility to public health decision-makers [23,24,25].

Although these results often are published in academic literature, decision-makers may not be aware that the studies exist, may not have access to the publication or the information contained therein, may not know how best to integrate the information into their decision-making processes, and/or may prefer to rely on scientific studies conducted by government, rather than non-governmental, researchers. Therefore, the existence of research, biosurveillance platforms, and official reporting mechanisms for infectious disease events does not necessarily indicate that these activities intersect and inform each other. 

As observed after the launch of the 2014 Global Health Security Agenda (GHSA) and associated action packages, much of the scientific information accessed by human and animal health officials and public health decision-makers was, and continues to be, generated by local and/or central diagnostic laboratories [26,27,28]. Continuing to address gaps in these capabilities can lead to significant advances in disease prevention, such as a recent response to Nipah virus in India [29]. However, different sectors (specifically, academic, industry, and non-profit organizations) comprise the science and technology communities that develop and provide the tools necessary for detection, characterization, and analysis of infectious disease events. The results of this basic and applied research are published in scientific articles and discussed at scientific conferences, and genetic sequences and other similar information are deposited in databases, many of which exist for various model systems (e.g., plants and animals) and microbes. The scientists who conduct these studies become experts in their fields, often having the skills to help understand the significance of unusual outbreaks with known pathogens and to characterize new pathogens that resemble the ones they study. For example, in 2003, researchers on three continents who studied known respiratory pathogens were able to identify the first member of the coronavirus family causing widespread pneumonia in humans, the SARS-CoV [2,3,30,31,32,33]. In addition, researchers who study insects contribute to the scientific knowledge about how mosquitoes and ticks transmit pathogens such as Zika virus and *Borrelia burgdorferi* (the causative agent of Lyme disease), respectively. However, the expertise of the independent researchers (i.e., researchers who are not embedded within public or veterinary health agencies) and the data they produce often are not included in the decision-making process for outbreak response, unless prior relationships exist between the researchers and the public health decision-makers and practitioners. 

The disconnect between research investment in human and animal health decision-making about infectious disease outbreaks and translation of data and expertise generated from research in the decision-making process may limit some early detection and response activities needed to prevent and control infectious disease outbreaks. This article describes the current state of scientific input in the public health decision-making process and highlights the different types of organizations involved in communicating scientific information before and during outbreaks. Based on the identified gaps, we consider approaches for promoting communication and trust-building between scientists (both governmental and non-governmental scientists) and policy-makers to ensure that existing data and knowledge can be brought to bear when preparing for, assessing, and responding to infectious disease incidents. Among these approaches, promoting objective, open communication among policy-makers and researchers (from the natural and social sciences) before, during, and after public health emergencies are critical for achieving the goals of the GHSA and related initiatives focused on reducing natural, accidental, and deliberate biological risks, frequently through the lens of One Heath.

## 2. Science Informing Global Health Security Decision Making

### Information Pathways and Decision-Making in Crises

The flow of scientific information into the global health security decision-making process relies on several key factors, including: (a) networks of experts who are familiar to decision-makers and trusted experts in their respective fields; (b) information that is accessible to organizations and individuals involved in public health response; (c) decision-makers’ ability to understand and evaluate scientific information; and (d) the use of scientific information by individual(s) responsible for assessing the public health situation and operational decisions. In this paper, we distinguish between scientific information (i.e., data) collected during an outbreak, and information generated by clinical or fundamental research prior to an outbreak and published in publicly-available literature, regardless of whether it is open access or available for a fee. In addition, we group together organizations involved in data generation, whether through research or epidemiological studies, which includes academic, industrial, non-profit, human and animal diagnostic, and government laboratories. We distinguish these scientists from public health decision-makers and practitioners, who play roles in policy-making and/or health response operations. All of these stakeholders are critical to the effective translation of data to public health emergency prevention, detection, and response.

Under non-emergency conditions, scientific and technical information usually is provided to policy and decision-makers of all levels (e.g., health and agricultural agencies, political leaders, and lawmakers) through a variety of means, including white papers, briefings, informal communication, published papers, and scientific conferences [34,35]. However, the flow of scientific information during emergencies is different, often reflecting the immediacy of the situation. The GHSA and International Health Regulations (IHR) provide a defined process, through guidance, for the generation and reporting of public health emergencies of potential international concern. No clear process exists for compiling and evaluating previously published scientific data to inform public health decision-making. Without trusted networks of experts and organizations that communicate scientific information to policy-makers objectively, interest groups which provide information selectively, may be the prevailing voice [36,37]. This situation may result in policy-makers developing trusted relationships with individuals and organizations with biases, which may limit objective and thorough examination of the human, animal, agricultural, or environmental health problem(s). At the same time, many researchers, though not all, do not engage with policy-makers because they do not believe they play a role in policy or decision-making and/or believe that decision-makers may not be willing to listen to their insights. This lack of engagement can limit the quality and objectivity of information being conveyed to decision-makers.

Limitations in effective translation of scientific information under emergency and non-emergency conditions determine its use in decision-making. For example, if information is perceived as partial (i.e., incomplete and/or highly uncertain) or people communicating the information are perceived as biased, decision-makers may question the utility of the data or disregard it completely. Similarly, data inconsistent with beliefs, traditions, or political agendas may be disregarded and/or discredited to maintain cultural and social realities. For example, a number of parents choose to not vaccinate their children for unsubstantiated reasons, including a disbelief in the necessity of the vaccines, perception that vaccines cause infections rather than prevent them, and belief that vaccines may cause autism [38]. Conversely, more complete data sets, more objective communication of the data, and clearer descriptions of the uncertainty of the data and analytic results may engender greater confidence in the information contributing to the decision-making process, especially if communicated effectively and appropriately for the audience.

In emergency situations, when timing and dynamics change, confidence in scientific information and advice is extremely important. Decision-makers frequently do not have time to identify and familiarize themselves with existing scientific information. Consequently, gaps in knowledge may develop, leading to uncertainty about the utility of scientific data. Similarly, uncertainty in known data also may lead decision-makers to question the utility of the scientific data. In addition, the process for sharing information with decision-makers may be cumbersome, inefficient, or nonexistent, all hampering scientifically-informed decision making. Although these limitations exist in non-emergency situations, they are exacerbated in emergencies. Therefore, during emergencies, decision-makers rely more on established relationships with experts for sourcing scientific information, which may include relevant knowledge and expertise (e.g., 2003 SARS-CoV outbreak) or only public health data, ignoring other sources (e.g., 2014–2016 West Africa EVD outbreak).

## 3. Key Gaps and Impediments to Science-Driven Decision Making

### 3.1. Data and Models

Incorporating social, natural, computational, and mathematical science analyses, including collection and characterization of specimens [39], into public health decision-making processes may help prevent future outbreaks of infectious diseases [40]. Full integration of information is difficult to achieve because of a lack of cross-pollination of disciplines and sectors [41]. Under-resourced individuals and organizations (including diagnostic and research laboratories, particularly in low-resource countries) may not have the capacity to conduct needed scientific assessments and communicate results to key audiences, which significantly limits the sharing and use of scientific information by researchers, health officials, and decision-makers. In addition, to evaluate the potential risk of emerging outbreaks, researchers and decision-makers must interpret new scientific findings from multidisciplinary studies and modeling data, which may vary in uncertainty based on the availability and veracity of the input data [42]. The relative lack of inter-disciplinary research and data analysis [43,44] in research of public health relevance contributes to these challenges of data interpretation and risk assessment. 

Scientific methodologies, such as ecological niche modeling and spatial regression analyses, could contribute to better situational awareness in public health crises [45,46,47,48]. Combining these analyses with existing case studies may improve outbreak prediction and prevention (e.g., recent assessments of mosquito vectors for Zika virus in the United States) [49]. These and other types of modeling approaches [50,51] help to identify the information needs for which little data exist by leveraging results from other studies and revealing key knowledge gaps that, if filled, could improve accuracy and reduce the uncertainty of computational models [42,44,52,53]. As data are generated and analytic capabilities improve, uncertainty associated with modeling and data analysis decreases. Therefore, investments in cross-disciplinary research on ecology, wildlife and domestic animals, human health, behavioral sciences, implementation science, and cultural anthropology are essential for understanding how humans interact with their environments and how these interactions facilitate the emergence of previously unknown, wildlife-derived pathogens in the human population [54,55,56,57,58]. Similar trends can be observed with integration of social and biomedical sciences research, where research on behavioral change can inform compliance with medical interventions [59,60,61]. Communicating these and other data clearly and concisely to public health decision-makers is important for translating research investments to public health practice [62].

### 3.2. Safety and Security

From a risk management and infection control perspective, data on the capability of nations to respond to emerging or re-emerging infectious disease events are incomplete and the local traditions that inform control measures generally are not integrated into formal public health responses [63,64,65,66,67,68,69]. However, these data play a key role in implementing measures that meet the objectives of the 2005 IHR, OIE (World Organization for Animal Health) Standards, and the GHSA objectives and Action Packages (https://ghsagenda.org/). In 2016, a Commission on a Global Health Risk Framework for the Future highlighted the neglected dimension of security in global health [70]. Still, the ability to protect scientists, healthcare providers, the community, and the environment from exposure to pathogens that could harm public health and safety often is overlooked. However, this situation may change through efforts such as the GHSA 2024 Framework [71].

Critical to successful outbreak prevention and management is recognizing the need to identify, test, and employ biosafety and biosecurity measures that are sustainable and adoptable in local conditions, account for local infrastructure, laws, and social structure, and prevent accidental and deliberate release of studied pathogens. Outbreak investigations for Ebola virus, Middle East Respiratory Syndrome coronavirus (MERS-CoV), and SARS-CoV demonstrated the need for locally effective biosafety measures that protect healthcare workers, diagnostic laboratory workers, and animal health workers from exposure to the outbreak viruses, and biosecurity measures that prevent access to pathogens by malicious actors. Applied research may identify measures that enhance current risk management efforts, such as laboratory and clinical biosafety, biosecurity, and biorisk management.

### 3.3. Cultural Awareness

Social science research can provide a better understanding of local culture and traditions, which strongly influence pathogen transmission and acceptance of medical and public health interventions [43]. During the 2014–2016 West African EVD outbreak, a lack of cultural awareness about local end-of-life traditions led to ineffective or unintentionally dangerous public health interactions and undocumented infections [72,73,74]. Eventually, the public health community began identifying approaches to communicate the risk of virus transmission from touching infected bodies, mitigate transmission events through culturally acceptable means, and reduce fear of death through appropriately chosen infection control methods (e.g., use of white, instead of black, body bags in West Africa [75]). Early engagement with communities and social scientists who study the culture, tradition, and linguistics of people from affected areas would help inform communication by decision-makers, mitigation strategies used by public health responders, and trust-building with the local population. Furthermore, leveraging the knowledge gained from these social science disciplines could enhance efforts to build trust among affected individuals rather than allow the persistence of distrust between local communities and foreign health workers [76,77]. Similar approaches should be used towards domestic and wild animal research, with animal and conservation ethics and local cultural and traditions considered.

Research involving bioethics and social equity helps scientists incorporate ethical principles in the design and conduct studies involving human participants affected by public health emergencies [78]. Such studies are critically important for research examining the effectiveness of candidate vaccines and medicines, understanding pathogen transmission and infection in natural settings, and testing non-pharmaceutical interventions for disease prevention and mitigation. Although such studies have been conducted for years, the U.S. National Academies of Science, Engineering, and Medicine highlighted research needs for preparedness and response to public health emergencies and associated bioethical considerations [79]. This focus on the bioethics of disaster research has prompted non-governmental and governmental organizations alike to evaluate challenges and identify solutions to promote ethical practices in research during public health emergencies. Building on this and other social science research can promote the development and implementation of clinical and public health research that takes into account the culture, society, and benefits to and needs of research participants.

## 4. Potential Solutions

The purpose of much of infectious disease research is to identify pharmaceutical and non-pharmaceutical approaches for preventing, detecting and monitoring, and responding to public health outbreaks of national, regional, and international concern. Data that could inform prevention, detection, and response activities are generated by several different types of studies, including mathematical modeling, epidemiological studies, environmental scanning, life-sciences studies (e.g., microbial genomics), and cultural anthropology. By integrating known, published data in these fields, considering key knowledge gaps and existing areas of uncertainty, scientists can assist public health responders and decision-makers in understanding initial cases and feasible infection control measures. However, the results of these investments have limited utility if they are not being conveyed to policy-makers before the occurrence of and during an emergency. Without this information, human and animal health officials and health care professionals are left to diagnose emerging outbreaks using sub-optimal approaches and driving response efforts that might be unnecessarily ineffective and promulgating distrust in health response efforts. 

Three approaches for addressing these gaps are communication, funding, and translation efforts. Although not explicitly described in this paper, international and national policies on data access and decisions made for political or national security purposes present additional challenges to fully informed decision-making. Some of the solutions described in this section may help reduce, but not eliminate, these challenges, highlighting the realities inherent in global governance of public health preparedness and response. Nevertheless, the proposed solutions could improve communication between researchers and decision-makers and enhance translation of research investments to inform public health practice before, during, and after emergencies.

### 4.1. Communication

Communication strategies that include better articulation and dissemination of existing scientific knowledge and modeling approaches (including their use, gaps, and limitations), their relevance to public health emergencies, and the inherent uncertainties in scientific assessment greatly would enhance high-level public health decision-making before, during, and after emergencies [34]. Better awareness about the types of public health decisions, associated information needs, operational constraints, time pressures of decision-makers, and limitations of current scientific knowledge would enable researchers to communicate scientific information more effectively. Understanding what is required of data and how data are best communicated in public health emergencies would provide researchers with the necessary operational context in which decision-makers must evaluate and base their decisions. With greater appreciation for the limitations of and information needs during the decision-making process, researchers can identify, integrate and distill data of greatest relevance to the specific emergency. 

Effective communication can be achieved through active interaction or written documents, and fostered in a variety of venues, including scientific conferences, science and society workshops, and governmental meetings. Although some of these efforts currently are used, their effectiveness can be improved by tailoring communication to the audience. Interactions cultivated among stakeholders before emergencies could promote the development of trusted relationships between decision-makers and scientists, which can serve as the foundation for reach-back during public health emergencies. In addition, interactions through networks, such as the GHSA and associated groups, could promote open lines of communication between governmental health security officials and scientists, facilitating information-sharing and enabling greater understanding of key questions with which decision-makers struggle [35]. These interactions are most effective if they are in place before crises occur and maintained after an emergency ends, which can lead to greater trust and familiarity between policy-makers and researchers and more opportunities for information-sharing in non-emergency situations. Throughout, promoting diversity of scientific expertise and experiences within these communications networks is critical for ensuring that policy-makers receive unbiased, objective information upon which to base their decisions.

### 4.2. Funding and Open Access

Research investments can enhance detection, characterization, assessment, and response to infectious diseases. However, several challenges exist with the current approaches: (1) limited funding is available for basic research for a majority of infectious diseases, particularly neglected tropical diseases and wildlife-associated, epizootic (animal only) diseases; (2) limited funding opportunities exist for multi-disciplinary, multi-sectoral research and education; (3) limited support is provided for social science research that is relevant to prevention and mitigation of infectious disease outbreaks; (4) research funding continuously changes for many infectious diseases, limiting the sustainability of individual efforts (e.g., the 2018 U.S. President’s proposed budget included funding cuts for efforts to prevent and respond to EVD outbreaks even as the 2018 outbreak in the DRC emerged [80,81]); (5) lack of communication from scientists to non-technical audiences, including policy-makers; and (6) lack of evaluation metrics for assessing the effectiveness of scientific input into the public health process.

To counter these challenges, government agencies, intergovernmental organizations, private funders, and philanthropic organizations should develop forward-looking, longer-term initiatives that support basic and applied research in a variety of natural and social sciences, and in efforts promoting integration and translation of scientific data to public health emergency prevention, detection, and response. Although not routinely done, proactive and stable funding for these and other scientific inquiries provides opportunities to increase the knowledge-base from which decision-makers can draw when considering appropriate infection control actions, a suggestion supported by several scientific organizations. For example, longer-term studies, such as those on New World hantaviruses, have produced a great deal of information relevant to public health [82], including changing infection prevalence with species richness [83], the preponderance of infected males [84], and the role of climatic changes in causing fluctuations in rodent reservoir populations and their links to localized, sporadic disease outbreaks. Although these studies were initiated as part of a reactive response to an acute outbreak—in this case, hantavirus pulmonary syndrome—in 1993–1994, the information produced addresses key knowledge gaps that can inform future outbreaks. Similarly, research supported during and after EVD outbreaks has generated data on wildlife reservoir hosts and people’s perceptions of health and healthcare practices, both of which could inform future outbreak assessments and response efforts. In addition, funders should establish a process through which the results and assessments can be communicated to public health decision-makers, leveraging the recent movement towards open access publication requirements. As a positive example, the Bill and Melinda Gates Foundation and The Wellcome Trust require all grantees to make their results publicly available, enabling access to various stakeholders, including decision-makers [85,86,87]. However, access to information does not ensure their use by decision-makers. In addition, new data protection laws may counteract these open access policies of funders and journals [88]. 

Specific approaches for promoting greater translation of research include scientific staff support for decision-makers, fellowship opportunities, cross-disciplinary cooperation, and strategic funding mechanisms (e.g., contracts and cooperative agreements). Scientists and funders should identify and support the integration and translation of science from multiple sectors, fields, and disciplines to identify key information gaps for global health security and provide the scientific foundation for assessing infectious disease risks. Funding support for training and fellowships can promote explicit scientific input into decision-making and encourage open sharing of data with other researchers and health officials. Researchers and research institutions should aim to shift the culture of data sharing by promoting the open sharing of data with public health practitioners as an academic product on par with publications, decreasing the potential for politicization or biased use of data [70]. Data sharing has been raised with H5N1 influenza A virus, Ebola virus, and Zika virus [89], and informed by efforts to promote equitable benefit of results from the sharing of data and samples from emerging outbreaks [90,91]. In 2014, the U.S. government passed the DATA (Data Transparency and Accountability) Act, which requires that data from federally-funded efforts be made open and available. The U.S. government’s DATA.gov website (https://www.data.gov/) is the platform that was developed to store and provide access to the datasets. In addition, agencies such as U.S. Geological Survey now have an ‘eternal data’ archive called Science Base (https://www.sciencebase.gov/catalog/). Despite these efforts, national policies restricting data access and sharing to foreign entities present new challenges to equitable and reciprocal data sharing, especially as biological research increasingly relies on data science approaches [92]. 

Approaches for improving communication between researchers and policy-makers, the funding landscape, and open access policies could help promote research that addresses key knowledge gaps in health security policy and practice, and translate funded research to global health decision-making.

### 4.3. Translation of Data

Looking forward, the 2024 Framework of the Global Health Security Agenda stresses communication, political and financial advocacy, and engagement of a more diverse set of stakeholders [71]. In part, these efforts intend to increase national-level investment and support for addressing shortcomings in human and animal health capabilities that currently limit effective prevention, detection, and response to public health emergencies of international concern. However, the new structure developed to progress towards these GHSA efforts could be enhanced further by including the research community as a critical stakeholder and focusing attention on data sharing among the research, public health, veterinary health, agriculture, and environmental health communities. Active engagement of the scientific arms of research and diagnostic entities (regardless of their sector, whether academic, industry, or government laboratories) with local and national public and veterinary health entities could enable better translation of scientific information to address public health needs. Recent calls for integrating veterinary and human health research to improve One Health efforts, including policy development and implementation, have been published [93,94]. Training on and implementation of data translation, improved strategies for communicating data and their associated limitations and/or statistical significance, and active participation of the scientific community in public health decision-making processes could reveal opportunities for leveraging data in an informative and timely manner. 

## 5. Conclusions

The global burden of infectious diseases and the increased attention to natural, accidental, and deliberate biological threats has resulted in scientific and financial investment in infectious disease research. However, the results of these studies often are not translated to prevention, detection, and response efforts. Furthermore, the needs, receptivity, and stakeholders involved in sharing scientific data before and during emergencies differ, which can lead to barriers towards research translation to human and animal health practice. Overcoming these barriers is necessary to prevent and mitigate emerging and re-emerging infectious diseases, including the recent epidemics caused by Zika virus in the Americas, Yellow fever virus (YFV) in Angola and the DRC, and Ebola virus in the DRC. The public health burden caused by influenza virus has led to the creation of WHO collaborating centers through which data on naturally circulating strains and results from basic and applied research are shared, informing influenza surveillance efforts. In addition, scientific data associated with the Zika virus disease outbreak has been placed in the public domain to facilitate prevention and control of the outbreak. However, these data sharing efforts are inconsistent across outbreaks, as demonstrated by the lack of similar data sharing practice in the YFV outbreak in Africa [95]. Furthermore, sharing of data is not the same as effective communication of the data.

Despite the increased investment for infectious disease research, significant knowledge gaps remain in host–pathogen interactions, urbanization and climactic influences on pathogen transmission, pathogen evolution, interactions between wild and domestic animals and humans, existence of unknown but naturally occurring pathogens, and other areas of interest. These knowledge gaps introduce uncertainty about what can be concluded from available data, which in turn can raise doubt in the utility of research results and validity of science-based conclusions during decision-making, especially in emergency situations. Advanced engagement and communication between researchers and policy-makers could help identify critical knowledge gaps that could reduce uncertainty levels and promote better trust between scientists and decision-makers. Encouraging and training scientists to recognize and translate research findings to public health decision-makers enhances these efforts. Effective communication and long-term funding are important for providing decision-makers with a clear understanding of what is known and what needs to be determined to improve prevention, detection, and response efforts of current and future outbreaks.
